# A rare genetic variant in the cleavage site of *prepro-orexin* is associated with idiopathic hypersomnia

**DOI:** 10.1038/s41525-022-00298-w

**Published:** 2022-04-12

**Authors:** Taku Miyagawa, Susumu Tanaka, Mihoko Shimada, Noriaki Sakai, Kotomi Tanida, Nozomu Kotorii, Tatayu Kotorii, Yu Ariyoshi, Yuji Hashizume, Kimihiro Ogi, Hiroshi Hiejima, Takashi Kanbayashi, Aya Imanishi, Azusa Ikegami, Yuichi Kamei, Akiko Hida, Yamato Wada, Masayuki Miyamoto, Masanori Takami, Hideaki Kondo, Yoshiyuki Tamura, Yukari Taniyama, Naoto Omata, Tomoyuki Mizuno, Shunpei Moriya, Hirokazu Furuya, Mitsuhiro Kato, Kayoko Kato, Jun Ishigooka, Kazuhito Tsuruta, Shigeru Chiba, Naoto Yamada, Masako Okawa, Koichi Hirata, Kenji Kuroda, Kazuhiko Kume, Naohisa Uchimura, Masaaki Kitada, Tohru Kodama, Yuichi Inoue, Seiji Nishino, Kazuo Mishima, Katsushi Tokunaga, Makoto Honda

**Affiliations:** 1 Sleep Disorders Project, Department of Psychiatry and Behavioral Sciences, Tokyo Metropolitan Institute of Medical Science, Tokyo, Japan; 2 Department of Human Genetics, Graduate School of Medicine, The University of Tokyo, Tokyo, Japan; 3 Department of Anatomy, Faculty of Medicine, Kansai Medical University, Osaka, Japan; 4 Genome Medical Science Project (Toyama), National Center for Global Health and Medicine, Tokyo, Japan; 5 Sleep and Circadian Neurobiology Laboratory, School of Medicine, Stanford University, Stanford, CA USA; 6 Department of Neuropsychiatry, Kurume University School of Medicine, Fukuoka, Japan; 7 Kotorii Isahaya Hospital, Nagasaki, Japan; 8 Ariyoshi Sleep Clinic, Fukuoka, Japan; 9 International Institute for Integrative Sleep Medicine (WPI-IIIS), University of Tsukuba, Ibaraki, Japan; 10 Ibaraki Prefectural Medical Center of Psychiatry, Ibaraki, Japan; 11 Department of Neuropsychiatry, Akita University Graduate School of Medicine, Akita, Japan; 12 Sleep Center, Kuwamizu Hospital, Kumamoto, Japan; 13 Department of Laboratory Medicine, National Center Hospital, National Center of Neurology and Psychiatry, Tokyo, Japan; 14 Kamisuwa Hospital, Nagano, Japan; 15 Department of Sleep-Wake Disorders, National Institute of Mental Health, National Center of Neurology and Psychiatry, Tokyo, Japan; 16 Department of Psychiatry, Hannan Hospital, Osaka, Japan; 17 Department of Neurology, Dokkyo Medical University, Tochigi, Japan; 18 Department of Psychiatry, Shiga University of Medical Science, Shiga, Japan; 19 Department of Psychiatry and Neurology, Asahikawa Medical University, Hokkaido, Japan; 20 Department of Neurology, Junwakai Memorial Hospital, Miyazaki, Japan; 21 Department of Nursing, Faculty of Health Science, Fukui Health Science University, Fukui, Japan; 22 Department of Neuropsychiatry, Faculty of Medical Sciences, University of Fukui, Fukui, Japan; 23 Department of Psychiatry, Tokyo Women’s Medical University School of Medicine, Tokyo, Japan; 24 Department of Neurology, Neuro-Muscular Center, National Omuta Hospital, Fukuoka, Japan; 25 Department of Neurology, Kochi Medical School, Kochi University, Kochi, Japan; 26 Department of Pediatrics, Yamagata University Faculty of Medicine, Yamagata, Japan; 27 Department of Pediatrics, Showa University School of Medicine, Tokyo, Japan; 28 Institute of CNS Pharmacology, Tokyo, Japan; 29 Department of Sleep Medicine, Shiga University of Medical Science, Shiga, Japan; 30 Japan Foundation for Neuroscience and Mental Health, Tokyo, Japan; 31 Department of Somnology, Tokyo Medical University, Tokyo, Japan; 32 Department of Stem Cell Biology, Institute of Molecular Genetics and Embryology, Kumamoto University, Kumamoto, Japan; 33 Department of Neuropharmacology, Graduate School of Pharmaceutical Sciences, Nagoya City University, Aichi, Japan; 34 Yoyogi Sleep Disorder Center, Neuropsychiatric Research Institute, Tokyo, Japan; 35 Seiwa Hospital, Institute of Neuropsychiatry, Tokyo, Japan

**Keywords:** Risk factors, Behavioural genetics, Sleep disorders

## Abstract

Idiopathic hypersomnia (IH) is a rare, heterogeneous sleep disorder characterized by excessive daytime sleepiness. In contrast to narcolepsy type 1, which is a well-defined type of central disorders of hypersomnolence, the etiology of IH is poorly understood. No susceptibility loci associated with IH have been clearly identified, despite the tendency for familial aggregation of IH. We performed a variation screening of the *prepro-orexin/hypocretin* and *orexin receptors* genes and an association study for IH in a Japanese population, with replication (598 patients and 9826 controls). We identified a rare missense variant (g.42184347T>C; p.Lys68Arg; rs537376938) in the cleavage site of *prepro-orexin* that was associated with IH (minor allele frequency of 1.67% in cases versus 0.32% in controls, *P* = 2.7 × 10^−8^, odds ratio = 5.36). Two forms of orexin (orexin-A and -B) are generated from cleavage of one precursor peptide, prepro-orexin. The difference in cleavage efficiency between wild-type (Gly-Lys-Arg; GKR) and mutant (Gly-Arg-Arg; GRR) peptides was examined by assays using proprotein convertase subtilisin/kexin (PCSK) type 1 and PCSK type 2. In both PCSK1 and PCSK2 assays, the cleavage efficiency of the mutant peptide was lower than that of the wild-type peptide. We also confirmed that the prepro-orexin peptides themselves transmitted less signaling through orexin receptors than mature orexin-A and orexin-B peptides. These results indicate that a subgroup of IH is associated with decreased orexin signaling, which is believed to be a hallmark of narcolepsy type 1.

## Introduction

Idiopathic hypersomnia (IH) is a rare heterogeneous disorder characterized by prolonged and disabling excessive daytime sleepiness (intolerable sleepiness in the daytime)^1^. Patients with narcolepsy type 1, which is a well-recognized type of central disorders of hypersomnolence, exhibit excessive daytime sleepiness, cataplexy (sudden loss of muscle tone in response to strong emotion), and abnormal rapid eye movement (REM) sleep. However, patients with IH do not display cataplexy or REM sleep abnormalities. The onset of IH is most frequently during adolescence or young adulthood^2^. IH is a rare disease and is estimated to affect approximately 0.005% of the general population^3^. However, the exact prevalence of IH remains unknown due to the absence of epidemiological studies. A high proportion of IH patients (26.9–39.1%) reported a family member with excessive daytime sleepiness^3–5^. The human leukocyte antigen (HLA) class II region is strongly associated with narcolepsy type 1 and type 2. Genome-wide association studies (GWAS) of narcolepsy type 1 have identified more than 10 genomic loci involved in immune and metabolic pathways^6–8^. When the 2009 influenza A (H1N1) pandemic was declared, the AS03-adjuvanted vaccine Pandemrix was used in several European countries. A significant association between the onset of narcolepsy type 1 and exposure to Pandemrix in children and adolescents was reported in Finland and Sweden^9^. A GWAS of Pandemrix-associated narcolepsy type 1 was performed, and *GDNF-AS1* was identified as a novel genetic factor^10^. Kleine-Levin syndrome (KLS) is a rare sleep disorder characterized by recurrent episodes of hypersomnia and is associated with cognitive disturbances and behavioral abnormalities. A GWAS of KLS found a significant association with variants in *TRANK1* region and links between KLS, circadian regulation, and bipolar disorder^11^. Meanwhile, no significant associations between IH and HLA have been observed^12^, and no other genetic variants associated with IH have been clearly identified^13^. Thus, the underlying genetic contribution to IH is poorly understood.

Orexin, also known as hypocretin, is a neuropeptide that regulates sleep-wake cycles and REM sleep^14–16^. Narcolepsy type 1 is caused by the loss of orexin-producing neurons in the hypothalamus, leading to low or undetectable levels of orexin-A in the cerebrospinal fluid (CSF)^17,18^. Rare missense mutations in *MOG* and *P2RY11* have been reported to be associated with narcolepsy type 1^19,20^. However, pathogenic mutations in *prepro-orexin* and *orexin receptor-1* (*OX1R*) and *-2* (*OX2R*) were not identified in patients with narcolepsy type 1, except for one rare severe case^18,21^. Previous studies have reported that postnatal cell death of orexin-producing neurons in narcolepsy is associated with cell type-specific autoimmune targeting^22,23^. Unlike patients with narcolepsy type 1, patients with IH show normal levels of orexin-A in the CSF. Therefore, orexin signaling system in IH has been believed to be normal^24^. Genetic variants in *prepro-orexin*, *OX1R*, and *OX2R* have not been studied in IH. Orexin is a master regulator of sleep, and we hypothesized that genetic variants may exist that affect the protein structure or its function.

In this study, we focused on rare missense and loss-of-function variants in *prepro-orexin*, *OX1R*, and *OX2R* to search for genetic factors of IH because no significant associations with common variants in these gene regions were observed in our GWAS^13^. IH has been reported to be clinically heterogeneous^25^. To identify rare genetic variants associated with IH, we conducted an association study between IH and genetic variants selected by variation screening in exons of these genes in the Japanese population, which is relatively homogeneous.

## Results

### A rare variant associated with idiopathic hypersomnia

A flow diagram of the study design is shown in Supplementary Fig. 1. For *prepro-orexin*, *OX1R*, and *OX2R*, variation screening of 195 patients with IH identified only one rare missense variant in *prepro-orexin* through the filtering process (see Methods) (g.42184347T>C; p.Lys68Arg; rs537376938). Then, we performed an association study between IH and p.Lys68Arg in 8380 controls and 440 patients with IH including the 195 patients with IH analyzed in the variation screening. The minor allele frequency (MAF) of p.Lys68Arg was 1.59% in the IH group and 0.30% in the control group, showing a significant association (*P* = 2.5 × 10^−6^, odds ratio = 5.40) (Table 1). We next assessed p.Lys68Arg in an independent set of 158 patients with IH and 1446 controls as a replication study. As a result, the p.Lys68Arg variant was replicated successfully (*P* = 6.0 × 10^−3^, odds ratio = 4.62) (Table 1). In the combined sample, the MAF of p.Lys68Arg was significantly higher in patients with IH than in controls, demonstrating a strong susceptibility to IH (MAF of 1.67% in cases versus 0.32% in controls, *P* = 2.7 × 10^−8^, odds ratio = 5.36) (Table 1). In contrast, no significant association between narcolepsy (type 1 and type 2) and p.Lys68Arg was observed (Supplementary Table 1). All patients with this mutation were heterozygous carriers. Clinical polysomnography (PSG) and multiple sleep latency test (MSLT) variables were available in a subset of our IH patients, and therefore, we compared these clinical data to find characteristic changes in patients with this orexin mutation (Table 2). Subjective sleepiness as evaluated with the Japanese version of the Epworth Sleepiness Scale (JESS) scores in unmedicated conditions and arousal index were nominally higher in the orexin mutation-positive patients (*P* = 0.049 and *P* = 0.046, respectively), suggesting that mutation-positive IH patients suffered more sleepiness and sleep instability similar to orexin-deficient narcolepsy type 1. Then, excluding patients who had periodic limb movement index (PLMI) ≥ 15 events per hour in adults, PLMI ≥ 5 events per hour in children, or apnea hypopnea index ≥5 events per hour, which may be associated with sleep problems at night, the remaining 317 patients with IH were analyzed for association with p.Lys68Arg. The variant also showed a significant association with these 317 patients (*P* = 8.5 × 10^−8^, odds ratio = 7.13) (Supplementary Table 2). We searched for MAFs of p.Lys68Arg in populations other than the Japanese population using whole-genome sequencing data of the Han Chinese Genomes Database (PGG.Han) and Genome Aggregation Database (gnomAD)^26,27^. The MAFs in the Han Chinese, European (non-Finnish), African, and European (Finnish) populations were 0.34%, 0.013%, 0%, and 0%, respectively. Next, we performed a principal component analysis (PCA) to determine whether population stratification or differences in the genetic background are present between the orexin mutation-positive and -negative IH groups. Genome-wide single nucleotide polymorphism (SNP) data in patients with IH (13 orexin mutation-positive patients and 116 orexin mutation-negative patients) and HapMap samples were utilized for the analysis. Our samples clustered in the JPT (Japanese in Tokyo)/CHB (Han Chinese in Beijing) cluster and separately from the CEU (Utah residents with Northern and Western European ancestry) and YRI (Nigeria Yoruba in Ibadan) clusters (Supplementary Fig. 2). When a PCA was conducted using our samples and JPT/CHB samples, the orexin mutation-positive and -negative IH samples seemed to be equally distributed in the JPT cluster (Supplementary Fig. 3). The result suggested that an effect of population stratification would be negligible in the samples. We also confirmed the absence of familial relationships among the mutation-positive patients using identity-by-descent (PIHAT) values (<0.1) calculated from the genome-wide SNP data.

**Table 1:** Significant association between rs537376938 (p.Lys68Arg) in *prepro-orexin* and IH.

Initial set				Replication set				Combined	
MAF (IH)	MAF (Control)	OR (95% CI)	*P*	MAF (IH)	MAF (Control)	OR (95% CI)	*P*	OR (95% CI)	*P*
1.59%	0.30%	5.40 (2.98–9.81)	2.5 × 10^−6^	1.89%	0.41%	4.62 (1.72–12.38)	6.0 × 10^−3^	5.36 (3.23–8.91)	2.7 × 10^−8^

rs537376938: p.Lys68Arg, g.42184347T>C, *IH* idiopathic hypersomnia, *MAF* minor allele frequency, *OR* odds ratio, *CI* confidence interval.

**Table 2:** Clinical characteristics of patients with IH in the present study.

Variable	IH	IH with mutation	IH without mutation	*P* value (mutation-positive vs. -negative)
Mean sleep latency (MSLT), min (*n*)	5.49 ± 0.18 (470)	4.52 ± 0.69 (18)	5.53 ± 0.18 (452)	0.18
Number of SOREMP (MSLT) (*n*)	0.35 ± 0.03 (491)	0.44 ± 0.24 (18)	0.34 ± 0.03 (473)	0.69
Sleep efficiency (PSG), % (*n*)	89.38 ± 0.50 (368)	90.72 ± 1.39 (17)	89.32 ± 0.52 (351)	0.37
Apnea hypopnea index (PSG), events/hour (*n*)	2.82 ± 0.18 (455)	3.60 ± 0.85 (19)	2.78 ± 0.18 (436)	0.37
Sleep latency (PSG), min (*n*)	11.45 ± 0.74 (453)	11.47 ± 2.92 (19)	11.44 ± 0.76 (434)	0.99
PLMI (PSG), events/hour (*n*)	1.11 ± 0.21 (370)	0.61 ± 0.31 (17)	1.14 ± 0.22 (353)	0.18
Arousal index (PSG), events/hour (*n*)	11.62 ± 0.30 (364)	13.64 ± 0.91 (16)	11.53 ± 0.31 (348)	0.046
JESS in unmedicated conditions (*n*)	15.73 ± 0.27 (313)	17.53 ± 0.82 (15)	15.64 ± 0.28 (298)	0.049
HLA-DQB1*06:02 positivity, % (*n*)	15.4 (592)	20.0 (20)	15.2 (572)	0.56

Means and standard errors are shown, except for HLA-DQB1*06:02 positivity.

*IH* idiopathic hypersomnia, *JESS* the Japanese version of the Epworth Sleepiness Scale, *MSLT* multiple sleep latency test, *PLMI* periodic limb movement index, *PSG* polysomnography, *SOREMP* sleep onset rapid eye movement period.

Large-scale GWASs using data from the UK biobank and 23andMe have identified that a common missense variant (g.55277539A>G; p.Ile308Val; rs2653349) in *OX2R* is significantly associated with self-reported napping^28^ (the Sleep Disorder Knowledge Portal website (http://www.sleepdisordergenetics.org)). The A allele of rs2653349 shows an association with more frequent napping during the day. Given the possibility of accumulation of genetic variants associated with daytime napping in the orexin pathway, we genotyped rs2653349 in patients with IH and controls. As a result, the A allele frequency of rs2653349 in the orexin mutation-positive IH group (15.0%) was significantly higher than that in the orexin mutation-negative IH group (4.7%) or control group (4.8%) (Table 3).

**Table 3:** Significant association between orexin mutation-positive IH and a common missense variant (rs2653349, p.Ile308Val) in *orexin receptor-2* (*OX2R*).

	A (Ile) allele frequency	G (Val) allele frequency	*P*	OR (95% CI)
Orexin mutation-positive IH (*n* = 20)	15.0%	85.0%		
Orexin mutation-negative IH (*n* = 532)	4.7%	95.3%	0.013^a^	3.58 (1.44-8.92)^a^
Control (*n* = 8380)	4.8%	95.2%	0.012^b^	3.47 (1.45-8.28)^b^

rs2653349: p.Ile308Val, g.55277539A>G, *IH* idiopathic hypersomnia, *OR* odds ratio, *CI* confidence interval.

^a^*P* value and OR were calculated for a comparison between orexin mutation-positive and -negative IH groups.

^b^*P* value and OR were calculated for a comparison between orexin mutation-positive IH and control groups.

Two missense variants, p.Arg168Trp (rs141639071) and p.Arg213His (rs200068306), were detected in *OX2R* by variation screening in patients with IH (Supplementary Table 3). These missense variants were registered in the Japanese Multi Omics Reference Panel (jMorp) (MAF of p.Arg168Trp = 0.11%, MAF of p.Arg213His = 0.03%). Only one IH patient each carried these missense variants, and thus, the two variants were excluded from further analyzes according to the work flow of this study. However, all in silico analyses estimated that p.Arg168Trp was damaging or deleterious, and therefore, we genotyped this mutation in IH patients not analyzed in the variation screening. No additional IH patients carried this variant, suggesting that p.Arg168Trp is not associated with IH, or the effect size of this variant may be weak even if an association between IH and p.Arg168Trp exists.

### Functional analysis of the identified variant

**Fig. 1:**
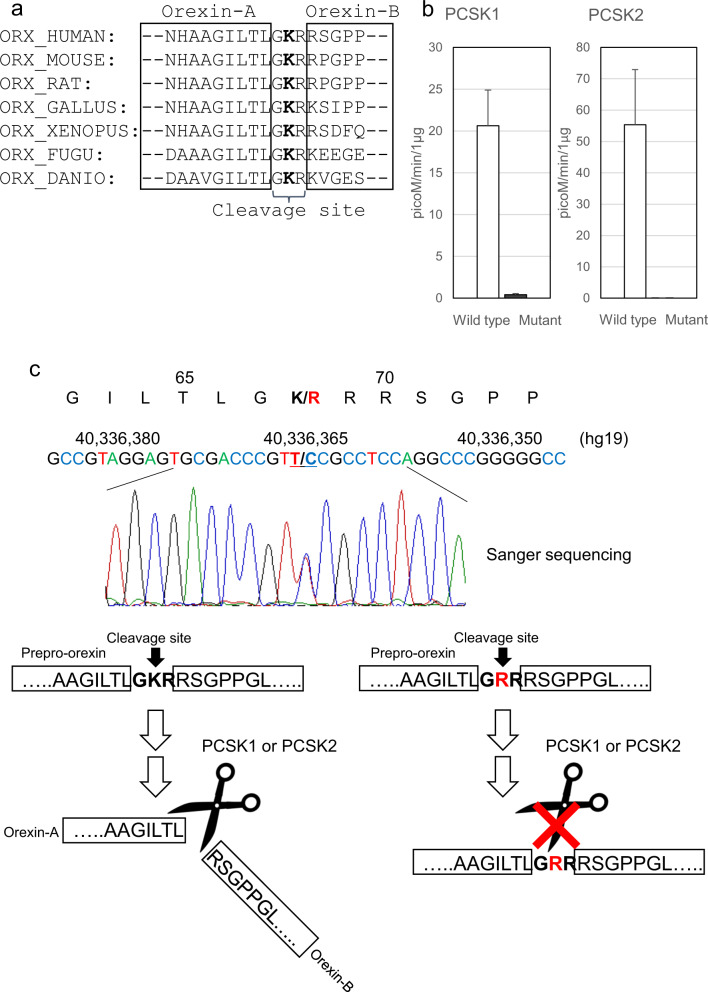
PCSK1 and PCSK2 enzyme activity against prepro-orexin peptides. **a** Alignment of part of the prepro-orexin (ORX) sequence from human (*Homo sapiens*), mouse (*Mus musculus*), rat (*Rattus norvegicus*), chicken (*Gallus gallus*), frog (*Xenopus laevis*), pufferfish (*Takifugu rubripes*), and zebrafish (*Danio rerio*). **b** Two peptides, wild-type orexin fragment (Wild type) and mutant orexin fragment (Mutant), were prepared for the enzyme activity analysis. All values are the mean ± standard deviation. **c** The p.Lys68Arg amino acid substitution was predicted to change the efficiency of prepro-orexin cleavage, which is catalyzed by PCSK1 or PCSK2.

**Fig. 2:**
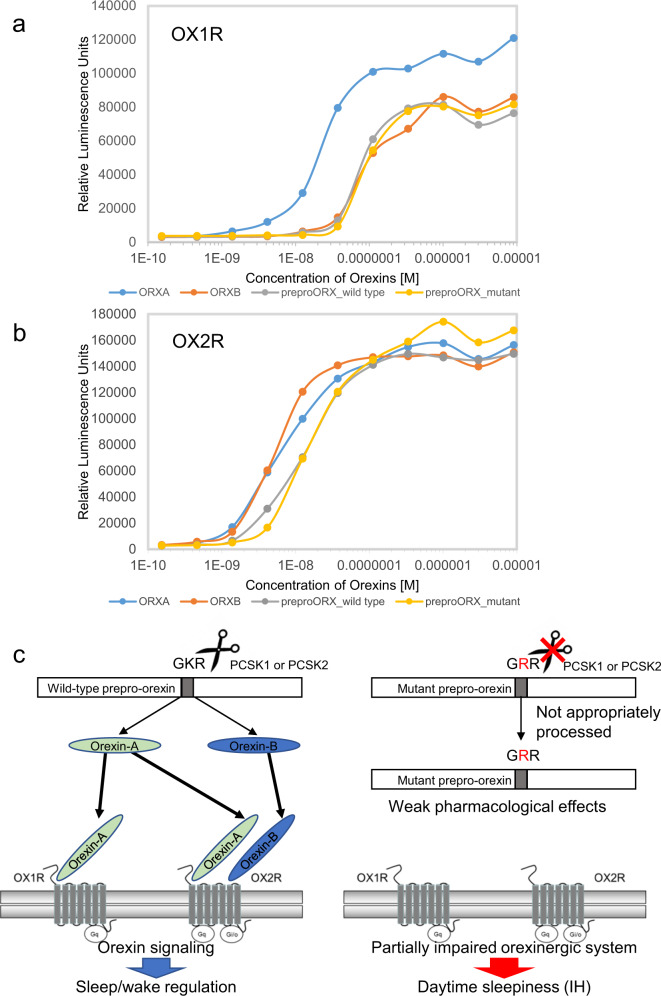
Pharmacological characterization of orexin peptides on orexin signaling through orexin receptors and schematic representation of orexin receptors. **a** (OX1R), **b** (OX2R) The ligand-induced β-arrestin recruitment system was utilized to assess pharmacological effects of human orexin-A (blue), orexin-B (red), full-length wild-type prepro-orexin (gray), and full-length mutant prepro-orexin (yellow). Light emission values were determined in duplicate. Circles on the lines indicate doses of the given peptides. **c** Orexin-A acts on both OX1R and OX2R, whereas orexin-B mainly acts on OX2R. The mutant form of prepro-orexin exhibited weak pharmacological effects on both OX1R and OX2R. OX1R is coupled exclusively to the Gq subclass of heterotrimeric G proteins, whereas OX2R may couple to Gi/o and/or Gq.

Orexin-A and orexin-B are cleaved from prepro-orexin. p.Lys68Arg is located in the cleavage site of prepro-orexin. GKR (Gly-Lys-Arg), which is the wild-type amino acid sequence of this cleavage site, is substituted with GRR (Gly-Arg-Arg) by the mutation. The Lys residue is conserved across almost all species that express prepro-orexin (Fig. 1a). This amino acid substitution was predicted to be damaging or deleterious by all in silico analyses (Supplementary Table 3). We hypothesized that the mutant amino acid sequence, GRR, affects the proteolytic cleavage efficiency of prepro-orexin. Two peptides, pLTLGKR-AMC (aminomethylcoumarin) (wild-type prepro-orexin) and pLTLGRR-AMC (mutant prepro-orexin), were prepared for an enzyme activity analysis. We examined proprotein convertase subtilisin/kexin (PCSK) type 1 and PCSK2 activity, which may process prepro-orexin^29^. In both the PCSK1 and PCSK2 assays, the mutant peptide was significantly less processed than the wild-type peptide (50-fold difference in PCSK1; 1100-fold difference in PCSK2) (Fig. 1b). The result suggested that large amounts of the mutant precursor prepro-orexin remain uncleaved in IH patients that carry p.Lys68Arg (Fig. 1c). Next, we investigated whether the prepro-orexin peptides have pharmacological effects on orexin signaling through OX1R and OX2R. The effects of orexin-A, orexin-B, wild-type prepro-orexin, and mutant prepro-orexin were evaluated using the ligand-induced β-arrestin recruitment system (Fig. 2a, b). Orexin-A induced the highest β-arrestin recruitment in the OX1R assay. The recruitment levels of the other three peptides were lower and similar to each other. In the OX2R assay, recruitment levels of both wild-type and mutant prepro-orexins were lower than those of orexin-A and orexin-B. These results indicated that the pharmacological effects of the prepro-orexin peptides on OX1R and OX2R were weak in terms of orexin signaling.

### HPLC analysis of CSF orexin-A

We collected a CSF sample from one of the 20 patients with IH who carried the mutant allele of p.Lys68Arg. Radioimmunoassay (RIA) measurement using a polyclonal antibody against orexin-A showed that the orexin-A level in CSF was normal at 227 pg/mL. Because degraded orexin-A fragments are largely found in the CSF and orexin-A production is partly perturbed by the mutation at the cleavage site of prepro-orexin, the degree of degradation of orexin-A peptide in the CSF was evaluated using high performance liquid chromatography (HPLC) followed by RIA, a method that has been recently developed. We compared the CSF orexin-A degradation pattern in this IH patient with the orexin mutation and another IH patient without this mutation. We detected two peaks in both CSF samples that are expected to be orexin-A fragments according to previous studies^30,31^. However, no significant differences in degradation in terms of the amount or separation pattern were observed (Supplementary Fig. 4).

## Discussion

The orexin pathway was not believed to contribute to the development of IH because the CSF orexin-A levels are normal in patients with IH^24^. However, considering the heterogeneity of IH and the importance of orexin in sleep–wake regulation, we hypothesized that some IH patients may carry variants associated with a dysfunction in the orexin signaling pathway and that detailed examination of orexin-related genes would be important. In this study, we found a significant association between IH and p.Lys68Arg, which is located in the cleavage site of prepro-orexin. Low cleavage efficiency of prepro-orexin with this mutation was confirmed. In addition, this form of prepro-orexin exhibited weak pharmacological effects on orexin signaling.

MAFs of p.Lys68Arg in populations other than Asian populations are very low, suggesting that the effect size of p.Lys68Arg would be low in other populations. A further replication analysis to confirm the association between IH and p.Lys68Arg should be performed in Asian populations. Rare variants show typically stronger stratification than common variants^32^. Although we performed a PCA, a family-based association study for replication would be more appropriate to exclude the possibility of rare variant stratification in a future study. In addition, the MAF of p.Lys68Arg in the Japanese controls was 0.32%. The sleep phenotype of these healthy individuals, not patients, with this mutation should be analyzed to determine whether they have specific sleep characteristics. In this study, sleep phenotype data for 398 healthy individuals were collected including the MSLT and the JESS. However, no individuals in this group have this mutation, and thus, we cannot currently address this issue.

MSLT, the gold standard for the diagnosis of central disorders of hypersomnolence, is criticized for poor precision in the differentiation of IH and narcolepsy type 2^33^. In this study, no significant association between narcolepsy type 2 and p.Lys68Arg was observed (Supplementary Table 1). Larger sample sizes of narcolepsy type 2 are required to provide a more reliable result about the association between them. In the future, prospective studies should be carried out to examine whether subjects with this orexin mutation tend to have a change in their diagnosis following MSLT.

Whole-exome sequencing is an efficient method to discover rare coding variants in all genes. However, according to the gnomAD, the sequence depth of p.Lys68Arg in the whole-exome sequencing is low because 36.9% of variant carriers have sequence depth in the 0–15 range. No variant carriers have sequence depth in the 0–15 range for whole-genome sequencing in the gnomAD. The GC content of the coding sequence of *prepro-orexin* is 73%. A high GC content region can affect sequence depth for exome sequencing^34^. GC content has less influence on sequence depth in whole-genome sequencing than whole-exome sequencing^35^. These observations suggest that Sanger sequencing or whole-genome sequencing is an appropriate method to search for coding variants in *prepro-orexin*.

GWASs in individuals of European ancestry of the UK Biobank and 23andMe have identified genetic loci associated with sleep duration, chronotype, or daytime sleepiness^36–38^. Because MAFs of p.Lys68Arg in populations other than Asian populations are extremely low, these GWASs did not identify p.Lys68Arg in *prepro-orexin* as a locus associated with sleep phenotypes. Therefore, p.Lys68Arg may be a population-specific variant associated with IH. In addition, our study suggested that intensive studies in populations with non-European ancestry may be useful for identification of rare variants with larger effect sizes that cannot be found in studies of populations with European ancestry.

GWAS for napping identified a common missense variant (p.Ile308Val; rs2653349) in *OX2R*^28^. No significant association of this variant with IH in general was found, but when we examined the association of this *OX2R* mutation in orexin mutation-positive IH patients, we found a significantly higher risk for IH in the population (Table 3). This result indicated that a combination effect of p.Ile308Val in *OX2R* and p.Lys68Arg in *prepro-orexin* may contribute to a higher predisposition to IH. In addition, a significant difference was found in the frequency of rs2653349 in *OX2R* between orexin mutation-positive IH and -negative IH groups. The result suggested that mutations in the orexin signaling pathway can be used as a marker for the IH subclass.

In this analysis, all patients with this mutation were heterozygous carriers. Because they have one normal allele (Lys), the phenotypes are considered to be different from those with narcolepsy. Decreased efficiency of prepro-orexin cleavage may lead to a decreased amount of the mature form of orexin A and B, resulting in difficulty in maintenance of wakefulness. A mutation in *OX2R* causes autosomal recessive canine narcolepsy in Dobermans^16^. Although heterozygotes do not show spontaneous narcoleptic symptoms, they exhibit unambiguous cataplexy-like symptoms after administration of drugs that act on cholinergic and monoaminergic systems^39^. This result suggests that these drugs can induce side effects in people with the p.Lys68Arg mutant allele. On the other hand, because nonpeptide orexin receptor agonists have been developed for the treatment of narcolepsy^40^, orexin receptor agonists may have a treatment effect in IH patients that carry p.Lys68Arg.

We assessed the pharmacological effects of four orexin peptides on orexin signaling (Fig. 2a, b). Orexin-B induced β-arrestin recruitment that was weaker than orexin-A for OX1R, whereas similar levels of orexin-A and -B were observed for OX2R. The results were consistent with a previous report^14^. In the OX1R analysis, both wild-type and mutant prepro-orexin peptides induced β-arrestin recruitment that was much lower than that of orexin-A for OX1R and that was similar to that of orexin-B. Both prepro-orexin peptides induced β-arrestin recruitment that was also lower compared with that of orexin-A and orexin-B. These results indicated that prepro-orexin peptides that are not appropriately processed cannot bind to or cannot function as ligands for OX1R or OX2R (Fig. 2c).

Orexin-A levels are generally measured with an RIA using a polyclonal antibody against orexin-A. Therefore, patients with IH carrying one mutant allele of the p.Lys68Arg substitution are expected to show a normal level of orexin-A in the CSF. In fact, we confirmed that the CSF orexin-A level of a patient with the orexin mutation was normal, although only one patient was analyzed. Degradation of orexin-A in one patient with the orexin mutation and another patient without the mutation was also evaluated with recently developed technology that utilizes HPLC and RIA; no significant differences between the samples were found (Supplementary Fig. 4). In this analysis, two peaks were detected in the CSF samples. Integrated analysis of the CSF peptidome and proteome found two peptide variants (residues 34–47 and 34–49) derived from the N-terminal part of orexin-A^30^. Both peptides contain cysteine moieties that form disulfide bonds (Cys39-Cys45 and Cys40-Cys47), providing stability to the peptide. Taken together, we suggest that either or both of the two peaks detected in our study are two peptides containing cysteine moieties in the N-terminal part of orexin-A (Supplementary Fig. 5). No cysteine moieties are present in orexin-B or in other regions of orexin-A. These would be mostly degraded in the CSF by other factors. Therefore, we assume that no abnormality is present in the degradation process in the patient with the mutant allele. However, considering that mutant prepro-orexin is less processed by PCSK1 and PCSK2 and that the regular RIA method largely detects degenerated fragments, the orexinergic system may be partially impaired in IH patients with the mutant allele newly identified in this study (Fig. 2c).

In the present study, we found that a portion of IH patients was associated with p.Lys68Arg in *prepro-orexin* and that this variant is an East Asian-specific mutation. Further association studies in other populations are needed to test whether other rare genetic variants in *prepro-orexin*, *OX1R*, and *OX2R* affect the development of IH. IH is probably a heterogeneous disease, suggesting that associations between IH and genes other than those associated with the orexin pathway may exist. Therefore, much effort, such as greatly increasing the sample sizes and whole-genome analyses, is required to identify more genetic variants associated with IH. International collaboration is necessary to collect sufficient DNA samples for future studies.

## Methods

### Subjects

The sample set was composed of 598 patients with IH and 9826 healthy controls in a Japanese population (initial set: 440 patients and 8380 controls; replication set: 158 patients and 1446 controls). Physician sleep specialists diagnosed the patients with IH according to the International Classification of Sleep Disorders third edition (ICSD-3)^1^. The statistical power of the replication study was calculated at the significance level of 0.05. We set the frequency of the susceptibility allele and the odds ratio to be 0.005 and 5.5, respectively, which were estimated from the initial stage^41^. The power was estimated to be ~0.8. Additional clinical details (PSG and MSLT) for a subset of IH patients for whom additional data were available were provided by several collaborative institutions. The reliability and validity of the Japanese version of the ESS have been confirmed in the Japanese population, and the JESS is used as a subjective measure of daytime sleepiness of patients^42^. We obtained scores of the JESS from 313 IH patients in unmedicated conditions. Genotyping for the *HLA-DQB1* locus in 592 patients with IH was conducted with a Luminex Multi-Analyte Profiling system with WAKFlow HLA typing kits (Wakunaga Pharmaceutical, Wakunaga, Hiroshima, Japan). Clinical and demographic characteristics of the patients are shown in Table 2 and Supplementary Table 4, respectively.

Regarding the 8380 controls in the initial sample set, we utilized data from subjects provided by the jMorp (https://jmorp.megabank.tohoku.ac.jp/201911/)^43,44^. The previous study provided demographic characteristics of the jMorp subjects^44^. We also studied 235 patients with narcolepsy type 2 and 514 patients with narcolepsy type 1. Narcolepsy type 2 exhibits REM sleep abnormalities and has the same symptoms as type 1 except for cataplexy. Because narcolepsy type 2 is a central disorder of hypersomnolence as defined in the ICSD-3^1^, we included narcolepsy type 2 as a case group in the present study. All subjects provided written informed consent. This study was approved by the Human Genome, Gene Analysis Research Ethics Committee of the University of Tokyo (G0910-(32)) and the Research Ethics Committee of Tokyo Metropolitan Institute of Medical Science (21-10).

### Variation screening, association studies, and statistical analyses

PCR and Sanger sequencing were used to screen the exons for genetic variants in *prepro-orexin*, *OX1R*, and *OX2R*. Sequences of amplification and sequencing primers are shown in Supplementary Table 5. This variation screening was performed in 195 patients with IH. Rare missense and loss-of-function variants with a MAF of <5% were selected. When two or more patients carried a specific variant, we conducted association studies between the variant and IH. The variant was genotyped with the Taqman method in 598 patients with IH (initial set: *n* = 440; replication set: *n* = 158), 235 patients with narcolepsy type 2, 514 patients with narcolepsy type 1, and 1446 healthy controls. Genetic data from 8380 subjects of the jMorp (whole-genome sequence) were included as controls. For in silico analyses, SIFT^45^, PolyPhen-2^46^, LRT^47^, Mutation Taster^48^, Mutation Assessor^49^, and PROVEAN^50^ were used to predict the pathogenicity of a variant by referring to a previous study^51^. SNPs (rs2653349 and rs141639071) in *OX2R* were genotyped with the Genome-Wide Human SNP Array 6.0, described later, and the Taqman method. Comparisons of frequencies between two groups were done using the Fisher exact test. Clinical characteristics were compared between mutation-positive IH patients and -negative IH patients using the t test or Fisher exact test, because the distributions of age, sex, and body mass index were not significantly different between the two groups. The significance level was set at *P* < 0.05 for all statistical analyses. Genomic positions refer to GRCh38/hg38.

### Genome-wide SNP typing

Genome-wide SNP typing was performed in 13 orexin mutation-positive IH patients, 116 orexin mutation-negative IH patients, and 420 healthy controls using the Genome-Wide Human SNP Array 6.0 according to the manufacturer’s protocols (Affymetrix Inc., Santa Clara, CA). Data generated by the array were analyzed with GeneChip Operating Software and Genotyping Console 4.0 (Affymetrix Inc). We included SNPs that showed genotyping call rates of >97%, MAFs of >5%, and *P* values of more than the threshold of the Hardy–Weinberg equilibrium (*P* > 0.001), which was evaluated using the Chi-2 test. We checked unknown familial relationships between subjects in this study with PIHAT values as calculated by PLINK 1.9^52^.

When calculating PIHAT values, linkage disequilibrium-based SNP pruning was conducted (*r*^2^ < 0.5). PCA was performed using the EIGENSTRAT program^53^ and PLINK 1.9 to evaluate population stratification or differences in genetic background among the study subjects genotyped with the SNP array. In the PCA, 45 JPT (Japanese in Tokyo, Japan), 90 CEU (Utah residents with Northern and Western European ancestry from the CEPH collection), 90 YRI (Nigeria Yoruba in Ibadan, Nigeria), and 45 CHB (Han Chinese in Beijing, China) were utilized that were derived from the International HapMap project.

### PCSK1 and PCSK2 enzyme activity assay

Human orexin-A and orexin-B peptides were purchased from the PEPTIDE Institute (Osaka, Japan). Human wild-type orexin-A cleavage site substrate pLTLGKR-AMC (pyroleucine–threonine–leucine–glutamine–lysine–arginine–aminomethylcoumarin) and human mutant orexin-A cleavage site substrate pLTLGRR-AMC (pyroleucine–threonine–leucine–glutamine–arginine–arginine–aminomethylcoumarin) were synthesized by the PEPTIDE Institute. Recombinant human PCSK1 and PCSK2 were purchased from R & D Systems (Minneapolis, MN). Enzyme activity studies were performed according to the manufacturer’s protocols. In brief, PCSK1 activity was measured as the rate of cleavage of 200 μM synthetic fluorogenic substrates, pLTLGKR-AMC or pLTLGRR-AMC, in 50 μL assay buffer (pH 6.0) including 25 mM MES, 5 mM CaCl_2_, 1% Brij-35, and 4 μg/mL recombinant human PCSK1 at 37 °C for 60 min. PCSK2 activity was measured as the rate of cleavage of 100 μM synthetic fluorogenic substrates, pLTLGKR-AMC or pLTLGRR-AMC, in 50 μL assay buffer (pH 5.0) including 50 mM NaOAc, 100 mM NaCl, 0.5% Brij-35, and 0.4 μg/mL recombinant human PCSK2 at 37 °C for 30 min. Cleaved fluorogenic substrate measurements were obtained eight times at 380-nm excitation and 460-nm emission using the endopoint mode in an EnSpire Workstation (PerkinElmer Co., Ltd., Waltham, MA).

### PathHunter β-arrestin assay

To test the effect of prepro-orexin peptides against OX1R and OX2R, we used the ligand-induced β-arrestin recruitment system. β-arrestins are ubiquitously expressed in all cell types and function in the desensitization of G-protein-coupled receptors (GPCR), control of GPCR intracellular trafficking, and activation of GPCRs in multiple signaling pathways^54–57^. PathHunter eXpress β-arrestin GPCR cells are engineered to co-express ProLink (PL)-tagged GPCR and Enzyme Acceptor (EA)-tagged β-arrestin. Activation of GPCR-PL induces β-arrestin-EA recruitment, resulting in complementation of the two β-galactosidase enzyme fragments (EA and PL). The resulting functional enzyme hydrolyzes substrate to generate a chemiluminescent signal. PathHunter eXpress β-arrestin human OX1R or human OX2R CHO-K1 cells (DiscoverX, Fremont, CA) were seeded in AssayComplete Cell Plating Reagent (DiscoverX) and incubated for 48 h at 37 °C and 5% CO_2_. Then, cells were treated with designated concentrations of human orexin-A, orexin-B, wild-type prepro-orexin, or mutant prepro-orexin for 90 min at 37 °C and 5% CO_2_. The resulting active enzymes hydrolyze the detection substrate (DiscoverX) to generate light, which was measured sequentially in duplicate using an EnSpire plate reader (PerkinElmer Co., Ltd.).

### Measurement of CSF orexin

Orexin-A levels in CSF were measured with a commercially available ^125^I RIA kit using a polyclonal antibody (RK-003-30, Phoenix Pharmaceuticals, Burlingame, CA). The orexin-A levels were defined as low (≤110 pg/mL), intermediate (>110 to ≤200 pg/mL), or normal (>200 pg/mL). To assess degradation of the orexin-A peptide in the CSF, 1-mL CSF samples were injected onto HPLC (Model 526 HPLC pump, Alltech) at a flow rate of 1 ml/min and linear gradient of 10–60% acetonitrile/0.1% trifluoroacetic acid over 40 min. Samples were separated by μBondapak C18 column 3.9 × 300 mm, 10 μM 125 A, (Waters Corporation, Milford, MA). The fractions were collected every minute. The orexin-A levels in each fraction were dried up using a vacuum centrifuge system (SpeedVac, Savant). After resuspension in 250 µl of deionized water, 100 µl was used in duplicates to measure orexin-A immunoreactive peaks in RIA. An in-house orexin-A antibody (1:200) and 125I-labeled orexin-A isotope (T-003-30, Phoenix Pharmaceuticals) were used. The detailed protocol was described in the methods section of a previous study^31^.

### Reporting summary

Further information on research design is available in the Nature Research Reporting Summary linked to this article.

## Supplementary information

SUPPLEMENTAL MATERIAL

Reporting Summary Checklist

## Data Availability

The genome-wide data can be accessed upon application to NBDC Human Database (https://humandbs.biosciencedbc.jp/en/) (NBDC research ID: hum0264, Japanese Genotype-phenotype Archive (JGA) accession number: JGAS000508). All remaining data are within the manuscript and its Supporting Information files.
