# Supplementary material for: A rare genetic variant in the cleavage site of prepro-orexin is associated with idiopathic hypersomnia
Source: NPJ Genom Med. 2022 Apr 12;7:29. doi: 10.1038/s41525-022-00298-w (PMC9005711; doi:10.1038/s41525-022-00298-w)
Supplement: Supplementary file 1 — SUPPLEMENTAL MATERIAL [file 41525_2022_298_MOESM1_ESM.pdf]

Supplementary Table 1. Association between p.Lys68Arg in *prepro-orexin* and narcolepsy.

	Allele frequency			
	C (Arg)	T (Lys)	OR (95% CI)	P value
Narcolepsy type 2 (n = 235)	0.64%	99.36%	2.03 (0.63-6.49)	0.19
Narcolepsy type 1 (n = 514)	0.29%	99.71%	0.92 (0.29-2.95)	1
Control (n = 9,826)	0.32%	99.68%		

rs537376938: p.Lys68Arg, g.42184347T>C

OR, odds ratio; CI, confidence interval

Supplementary Table 2. Significant association between p.Lys68Arg in *prepro-orexin* and IH patients with PLMI <15 events per hour in adults, PLMI <5 events per hour in children, and apnea hypopnea index <5 events per hour.

	Allele frequency			
	C (Arg)	T (Lys)	OR (95% CI)	P
IH<br>(n = 317)	2.21%	97.79%	7.13<br>(3.97-12.81)	8.5×10^{-8}
Control<br>(n = 9,826)	0.32%	99.68%		

IH, idiopathic hypersomnia

PLMI, periodic limb movement index

Supplementary Table 3. Missense variants in three genes were detected with variation screening in patients with IH.

Chromosome	Position_hg19	rs number	Gene_annotation	*in silico* analyses							The Japanese Multi Omics Reference Panel (jMorp)			
				Polyphen2 HumDiv	SIFT	LRT	PROVEAN	Mutation Taster	Mutation Assessor	CADD	Ref_allele	Frequency Ref_allele	Alt_allele	Frequency Alt_allele
chr17	40336365	rs537376938	HCRT:ENST00000293330:NON_SYNONYMOUS_CODING:K68R	Probably damaging	Damaging	Deleterious	Deleterious	Disease causing	Medium	25.4	T	0.9967	C	0.0033
chr6	55120033	rs141639071	HCRTR2:ENST00000370862:NON_SYNONYMOUS_CODING:R168W	Probably damaging	Damaging	Deleterious	Deleterious	Disease causing	Medium	25.9	C	0.9989	T	0.0011
chr6	55120169	rs200068306	HCRTR2:ENST00000370862:NON_SYNONYMOUS_CODING:R213H	Benign	Tolerated	Neutral	Neutral	Polymorphism	Neutral	7.007	G	0.9997	A	0.0003

Each patient with IH carried a mutant allele of rs141639071 or rs200068306.

IH, idiopathic hypersomnia

Supplementary Table 4. Demographic characteristics of patients with IH in the present study.

Variable	IH	IH with mutation	IH without mutation	P values (mutation-positive vs. -negative)
Age (*n*)	26.6 ± 10.2 (598)	27.1 ± 7.0 (20)	26.6 ± 10.3 (578)	0.77
Sex, % female (*n*)	52.8 (598)	40.0 (20)	53.3 (578)	0.26
BMI (*n*)	21.6 ± 3.4 (522)	22.1 ± 3.0 (19)	21.6 ± 3.4 (503)	0.49

The means and standard deviations are presented, except for sex.

BMI, body mass index; IH, idiopathic hypersomnia

Supplementary Table 5. Amplification and sequencing primers.

1. Amplification (PCR) primers

Gene	Exon	Forward primer	Reverse primer
prepro-orexin	1	cagacaagggggtgatcagg	tttcttccagccctctgagc
	2	cagccagaaaggagtgcag	cttccaggcccagctgtc
orexin receptor-1 (OX1R)	1	tgcaacccaggtctgtcttc	agagccacacccatcctagt
	2	ggctttccctggggattgaa	agtgtgtggggttgtgtctg
	3	tcaggatttgcactgccctg	tggtaggagccagtctaggg
	4	ctgctcctaggccttgcttt	ttgacgtgtctctgggcttc
	5	tccttttgcccatctccacc	agaagagaaatggcctgccc
	6	tcaggtacagaaggccagga	gcaggtacatcctcacccac
	7	cccctcataggcagcttgg	ctctggaaggaggatgggga
orexin receptor-2 (OX2R)	1	ttcttcagcttcagctctcc	ggagggaggggtctctttag
	2	acggcacagccttcaattat	aatttaccataagggccatcg
	3	tggcagctttgaatttgctt	gcccagctttacaatggcta
	4	gcactttgaagaaaagcattga	cacagacaaaatatttggaaggaa
	5	ttttctaattacttcccaaagtg	gaaagtttagatttttctctggctta
	6	ggacaggagtcagaccatcc	cctcatatagttgaagagtgttcattg
	7	tagtttggctcaagggagca	tgaaactaaaagtgaaatagcctgaa

2. Sanger sequencing primers

Gene	Exon	Forward primer	Reverse primer
prepro-orexin	1	cagacaagggggtgatcagg	tttcttccagccctctgagc
	2 (first half)	gacagccagaaaggagtgca*	tcacacgaatggagactcgtc*
	2 (second half)	gacgctactgctgctgct*	cttccaggcccagctgtc
orexin receptor-1 (OX1R)	1	tgcaacccaggtctgtcttc	agagccacacccatcctagt
	2	ggctttccctggggattgaa	agtgtgtggggttgtgtctg
	3	tcaggatttgcactgccctg	tggtaggagccagtctaggg
	4	ctgctcctaggccttgcttt	ttgacgtgtctctgggcttc
	5	tccttttgcccatctccacc	agaagagaaatggcctgccc
	6	tcaggtacagaaggccagga	gcaggtacatcctcacccac
	7	cccctcataggcagcttgg	ctctggaaggaggatgggga
orexin receptor-2 (OX2R)	1	ttcttcagcttcagctctcc	ggagggaggggtctctttag
	2	acggcacagccttcaattat	aatttaccataagggccatcg
	3	tggcagctttgaatttgctt	gcccagctttacaatggcta
	4	gcactttgaagaaaagcattga	cacagacaaaatatttggaaggaa
	5	ttttctaattacttcccaaagtg	gaaaaggcaggctatttcattt*
	6	ggacaggagtcagaccatcc	cctcatatagttgaagagtgttcattg
	7	tagtttggctcaagggagca	tgaaactaaaagtgaaatagcctgaa

Sequencing primer marked with an asterisk (*) are different from PCR primers of the corresponding exon.

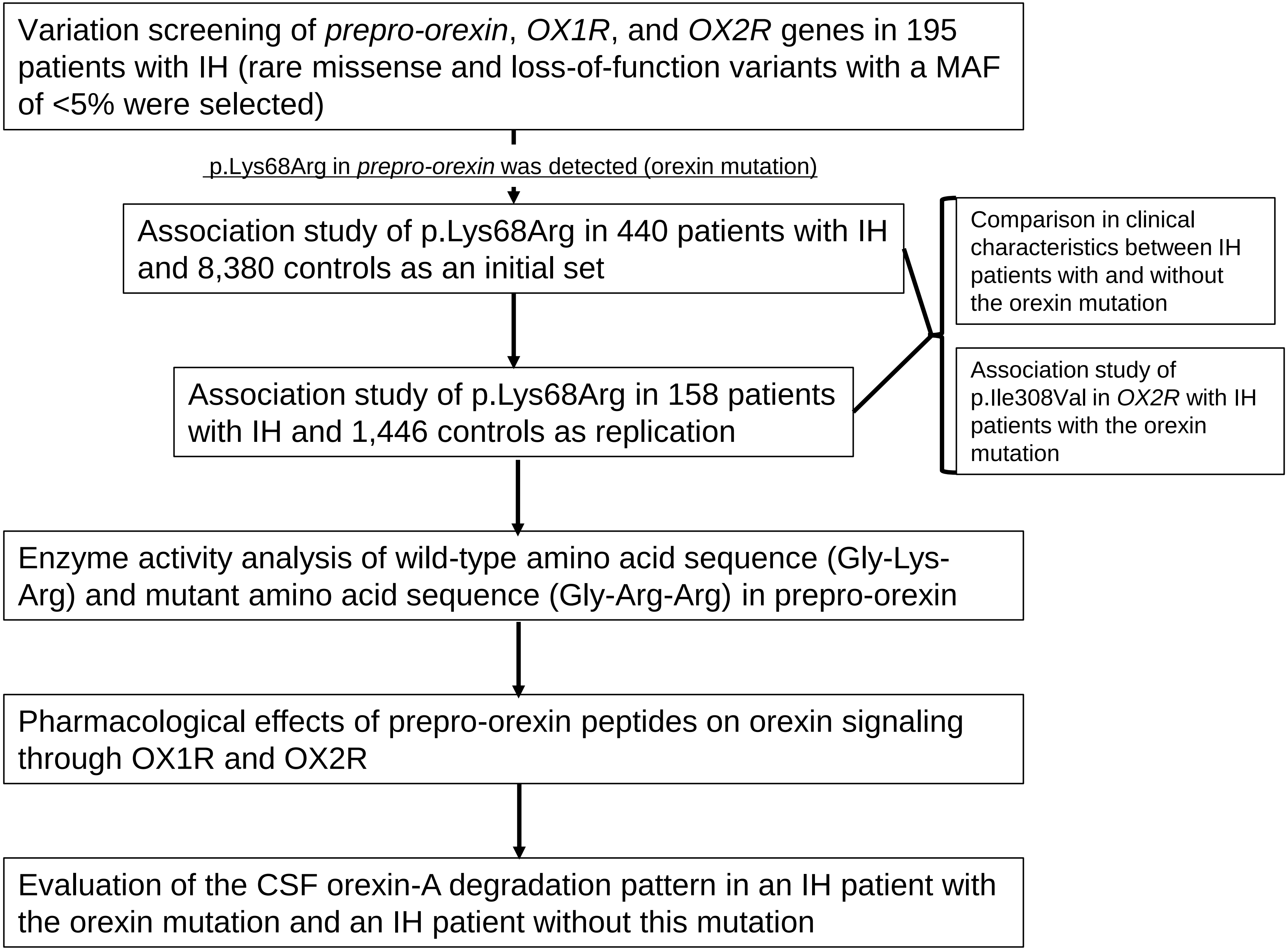

Supplementary Fig. 1. Flow diagram of the study design.
CSF, cerebrospinal fluid; IH, idiopathic hypersomnia; MAF, minor allele frequency; OX1R, orexin receptor-1; OX2R, orexin receptor-2

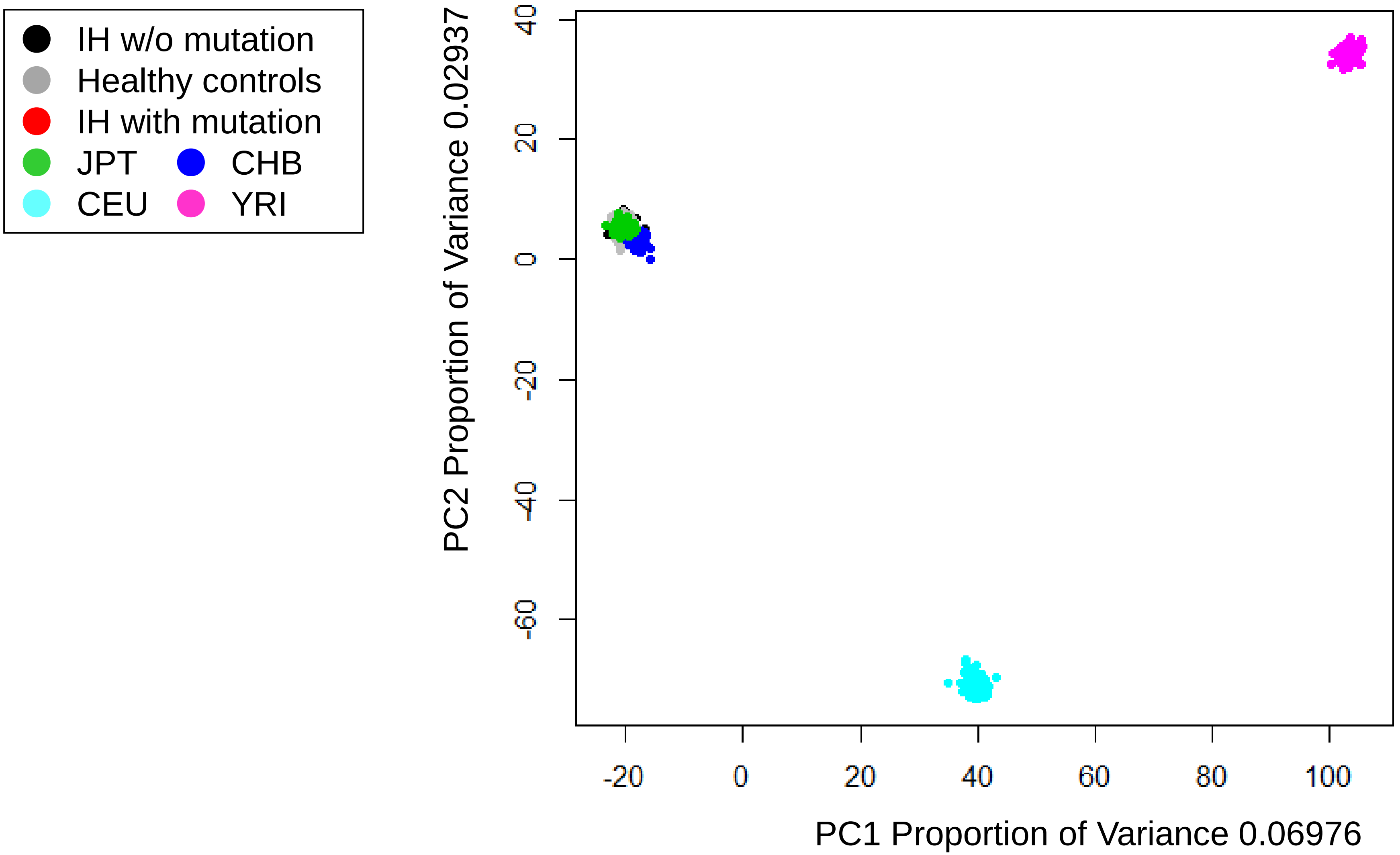

Supplementary Fig. 2. Principal component analysis (PCA).
We performed a PCA with 13 orexin mutation-positive IH patients (IH with mutation), 116 orexin mutation-negative IH patients (IH w/o mutation), 420 healthy controls, 45 JPT (Japanese in Tokyo, Japan), 90 CEU (Utah residents with Northern and Western European ancestry from the CEPH collection), 90 YRI (Nigeria Yoruba in Ibadan, Nigeria), and 45 CHB (Han Chinese in Beijing, China).

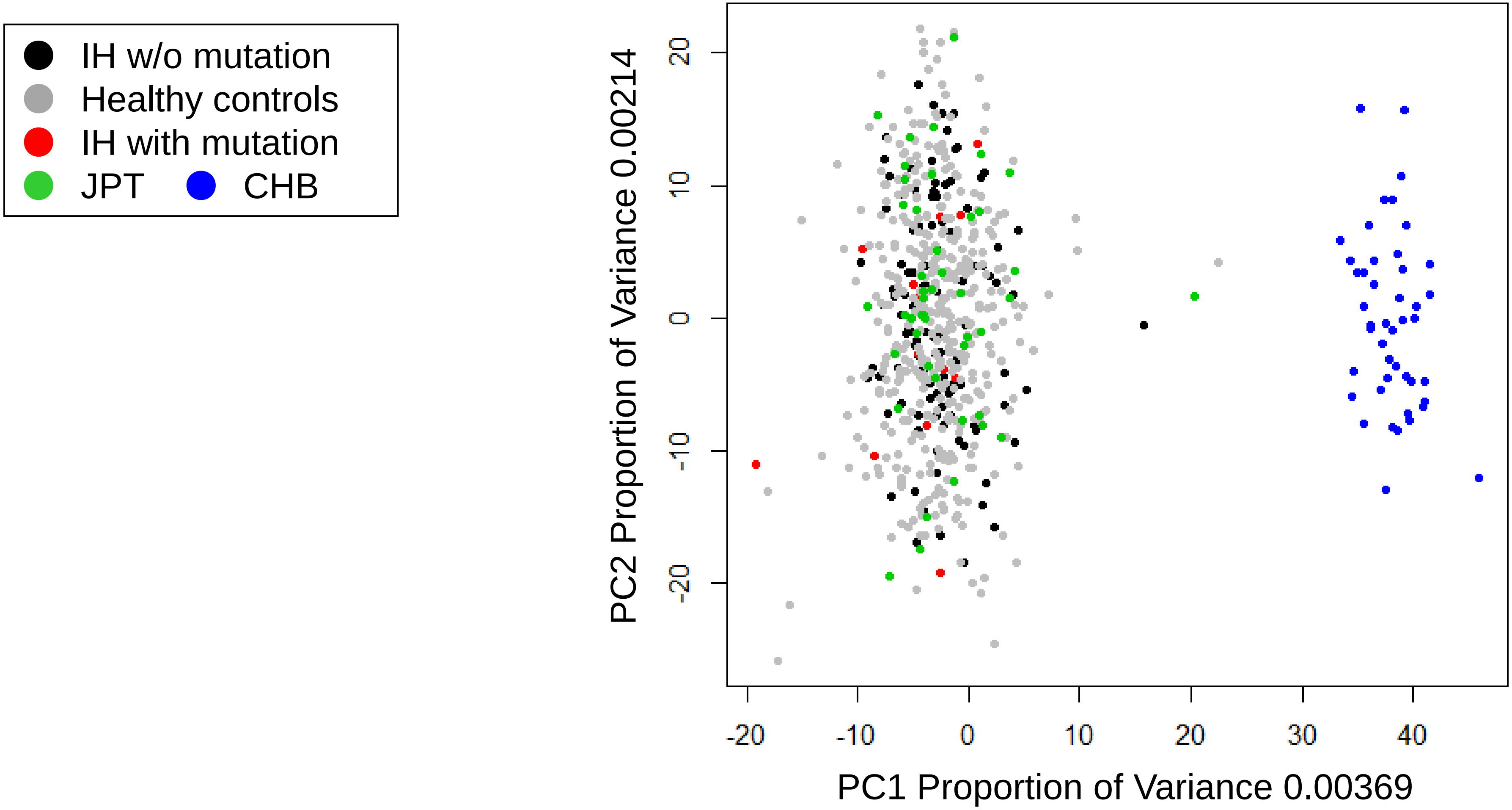

Supplementary Fig. 3. Principal component analysis (PCA) in our samples and East Asian populations.
We performed a PCA with 13 orexin mutation-positive IH patients (IH with mutation), 116 orexin mutation-negative IH patients (IH w/o mutation), 420 healthy controls, 45 JPT (Japanese in Tokyo, Japan), and 45 CHB (Han Chinese in Beijing, China).

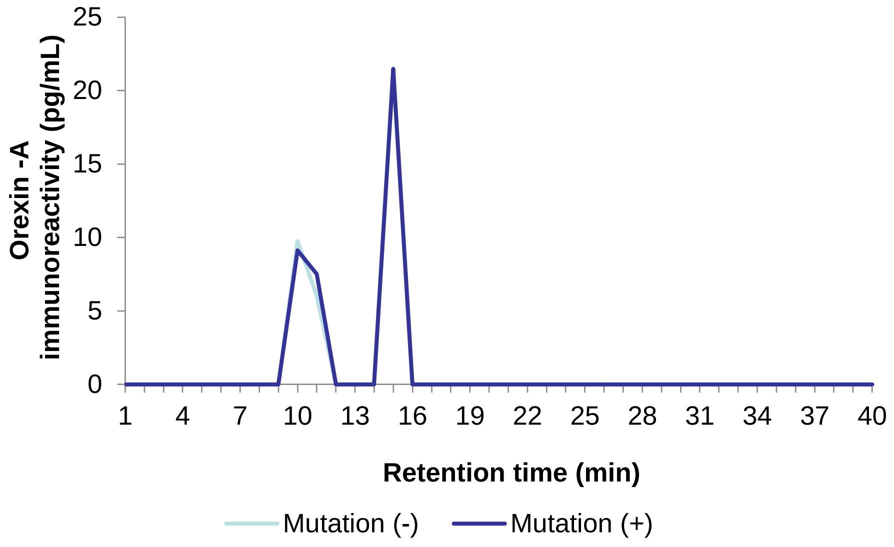

Supplementary Fig. 4. HPLC (high performance liquid chromatography) separation pattern of CSF (cerebrospinal fluid) samples from IH (idiopathic hypersomnia) patients with and without the mutation.

CSF was separated and detected with HPLC and RIA (radioimmunoassay) as previously reported.

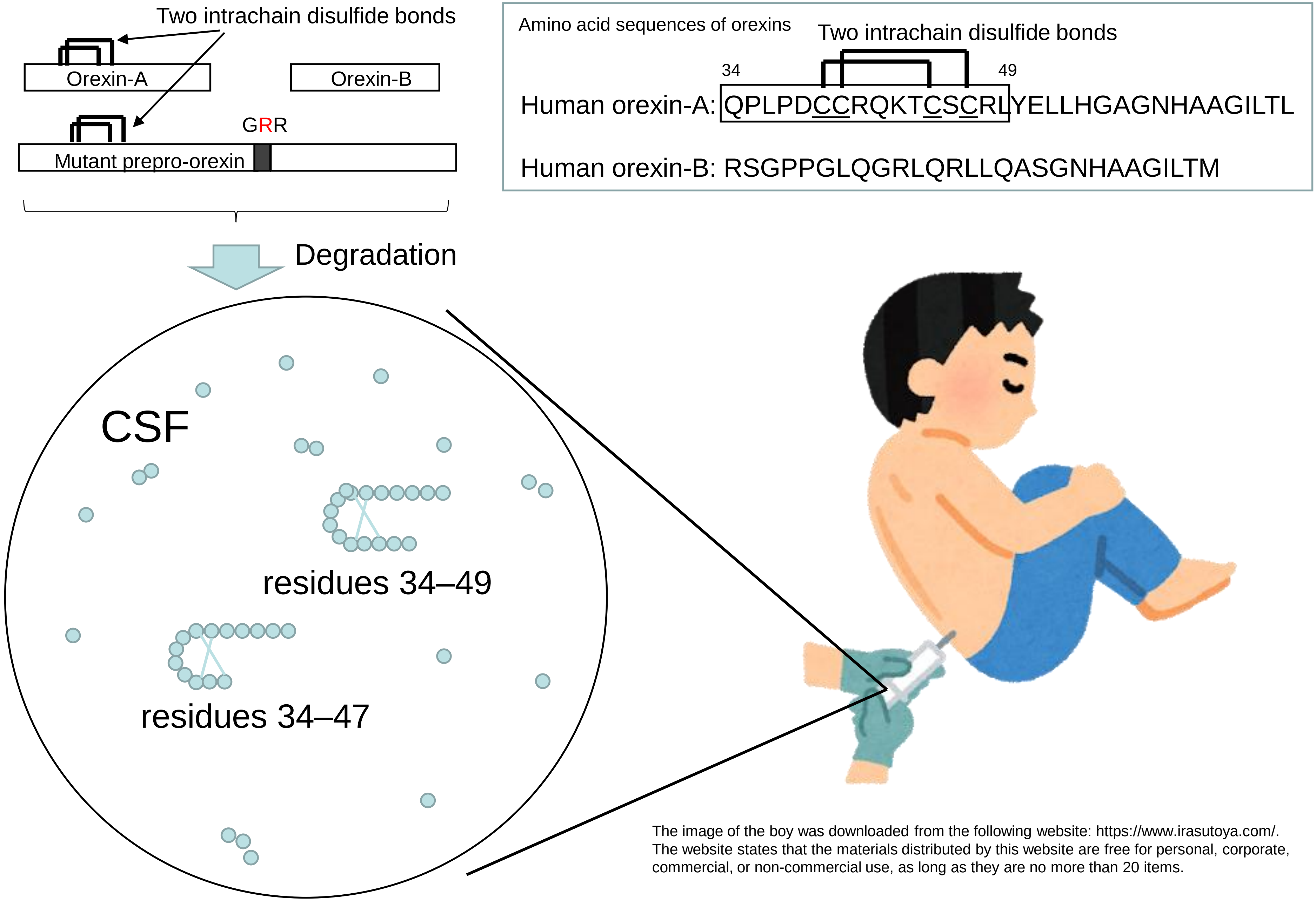

Supplementary Fig. 5. The two intrachain disulfide bonds.
Two peptide variants (residues 34–47 and 34–49) derived from orexin-A or prepro-orexin can be detected in the CSF (cerebrospinal fluid).
