# Supplementary material for: A rare genetic variant in the cleavage site of prepro-orexin is associated with idiopathic hypersomnia
Source: NPJ Genom Med. 2022 Apr 12;7:29. doi: 10.1038/s41525-022-00298-w (PMC9005711; doi:10.1038/s41525-022-00298-w)
Supplement: Supplementary file 2 — Reporting Summary Checklist [file 41525_2022_298_MOESM2_ESM.pdf]

nature portfolio

Corresponding author(s): Taku Miyagawa

Last updated by author(s): Mar 4, 2022

Reporting Summary

Nature Portfolio wishes to improve the reproducibility of the work that we publish. This form provides structure for consistency and transparency in reporting. For further information on Nature Portfolio policies, see our Editorial Policies and the Editorial Policy Checklist.

Statistics

For all statistical analyses, confirm that the following items are present in the figure legend, table legend, main text, or Methods section.

n/a	Confirmed	
☐	☒	The exact sample size (n) for each experimental group/condition, given as a discrete number and unit of measurement
☐	☒	A statement on whether measurements were taken from distinct samples or whether the same sample was measured repeatedly
☐	☒	The statistical test(s) used AND whether they are one- or two-sided<br>*Only common tests should be described solely by name; describe more complex techniques in the Methods section.*
☐	☒	A description of all covariates tested
☐	☒	A description of any assumptions or corrections, such as tests of normality and adjustment for multiple comparisons
☐	☒	A full description of the statistical parameters including central tendency (e.g. means) or other basic estimates (e.g. regression coefficient) AND variation (e.g. standard deviation) or associated estimates of uncertainty (e.g. confidence intervals)
☐	☒	For null hypothesis testing, the test statistic (e.g. F, t, r) with confidence intervals, effect sizes, degrees of freedom and P value noted<br>*Give P values as exact values whenever suitable.*
☒	☐	For Bayesian analysis, information on the choice of priors and Markov chain Monte Carlo settings
☐	☒	For hierarchical and complex designs, identification of the appropriate level for tests and full reporting of outcomes
☐	☒	Estimates of effect sizes (e.g. Cohen's d, Pearson's r), indicating how they were calculated

Our web collection on statistics for biologists contains articles on many of the points above.

Software and code

Policy information about availability of computer code

Data collection	No software was used for data collection.
Data analysis	DNA samples were genotyped using Affymetrix Genome-Wide Human SNP Array 6.0 platform. The genotype calling was conducted using GeneChip Operating Software (GCOS) and Genotyping Console 4.0 (Affymetrix), which employs the Birdseed version 2 (Birdseed v2) genotype calling algorithm. We used EIGENSTRAT program and PLINK 1.9 to analyze genome-wide SNP data. For in silico analyses, SIFT, PolyPhen-2, LRT, Mutation Taster, Mutation Assessor, and PROVEAN were used to predict the pathogenicity of each variant.

For manuscripts utilizing custom algorithms or software that are central to the research but not yet described in published literature, software must be made available to editors and reviewers. We strongly encourage code deposition in a community repository (e.g. GitHub). See the Nature Portfolio guidelines for submitting code & software for further information.

Data

Policy information about availability of data

All manuscripts must include a data availability statement. This statement should provide the following information, where applicable:

- Accession codes, unique identifiers, or web links for publicly available datasets
- A description of any restrictions on data availability
- For clinical datasets or third party data, please ensure that the statement adheres to our policy

The genome-wide data can be accessed upon application to NBDC Human Database (https://humandbs.biosciencedbc.jp/en/) (NBDC research ID: hum0264, Japanese Genotype-phenotype Archive (JGA) accession number: JGAS000508). All remaining data are within the manuscript and its Supporting Information files.

Field-specific reporting

Please select the one below that is the best fit for your research. If you are not sure, read the appropriate sections before making your selection.

☒ Life sciences ☐ Behavioural & social sciences ☐ Ecological, evolutionary & environmental sciences

For a reference copy of the document with all sections, see nature.com/documents/nr-reporting-summary-flat.pdf

Life sciences study design

All studies must disclose on these points even when the disclosure is negative.

Sample size	Sample set was composed of 598 patients with idiopathic hypersomnia and 9,826 healthy controls in a Japanese population (initial set: 440 patients and 8,380 controls; replication set: 158 patients and 1,446 controls). No sample size calculation was performed for the initial set. The statistical power of the replication study was calculated at the significance level of 0.05. We set the frequency of the susceptibility allele and the odds ratio to be 0.005 and 5.5, respectively, which were estimated from the initial stage. The power of the replication study was estimated to be approximately 0.8. We also studied 235 Japanese patients with narcolepsy type 2 and 514 Japanese patients with narcolepsy type 1.
Data exclusions	In the genome-wide SNP typing, we included SNPs that showed genotyping call rates of >97%, MAFs of >5%, and P-values of more than the threshold of the Hardy-Weinberg equilibrium (P > 0.001), which was evaluated using the Chi-2 test. We checked unknown familial relationships between subjects in this study with PIHAT values as calculated by PLINK 1.9. When calculating PIHAT values, linkage disequilibrium-based SNP pruning was conducted (r2 < 0.5).
Replication	We performed a replication study (replication set: 158 patients with idiopathic hypersomnia and 1,446 controls) and confirmed that the result was replicated.
Randomization	This is not relevant for the present study. No group allocation was performed.
Blinding	This is not relevant for the present study. No group allocation was performed.

Reporting for specific materials, systems and methods

We require information from authors about some types of materials, experimental systems and methods used in many studies. Here, indicate whether each material, system or method listed is relevant to your study. If you are not sure if a list item applies to your research, read the appropriate section before selecting a response.

Materials & experimental systems

n/a	Involved in the study	
☐	☒	Antibodies
☒	☐	Eukaryotic cell lines
☒	☐	Palaeontology and archaeology
☒	☐	Animals and other organisms
☐	☒	Human research participants
☒	☐	Clinical data
☒	☐	Dual use research of concern

Methods

n/a	Involved in the study	
☒	☐	ChIP-seq
☒	☐	Flow cytometry
☒	☐	MRI-based neuroimaging

Antibodies

Antibodies used	1. Orexin-A levels in CSF were measured with a commercially available 125I RIA kit using a polyclonal antibody (RK-003-30, Phoenix Pharmaceuticals, Burlingame, CA).<br>2. An in-house orexin-A antibody (1:200) and 125I-labeled orexin-A isotope (T-003-30, Phoenix Pharmaceuticals) were used to assess degradation of the orexin-A peptide.
Validation	1. Website: https://www.phoenixpeptide.com/products/view/Assay-Kits/RK-003-30<br>2. Sakai, N. et al. HPLC analysis of CSF hypocretin-1 in type 1 and 2 narcolepsy. Scientific Reports volume 9, Article number: 477 (2019), https://doi.org/10.1038/s41598-018-36942-8.

Human research participants

Policy information about studies involving human research participants

Population characteristics	Sample set was composed of 598 patients with idiopathic hypersomnia and 9,826 healthy controls in a Japanese population (initial set: 440 patients and 8,380 controls; replication set: 158 patients and 1,446 controls). In the present study, 235

	Japanese patients with narcolepsy type 2 and 514 Japanese patients with narcolepsy type 1 were also analyzed. Physician sleep specialists diagnosed the patients according to the International Classification of Sleep Disorders third edition (ICSD-3).
Recruitment	Samples and clinical data from patients with central disorders of hypersomnolence and 1,446 controls in the replication set were provided by participating centers. Regarding the 8,380 controls in the initial sample set, we utilized public data from subjects provided by the jMorp (https://jmorp.megabank.tohoku.ac.jp/201911/). There is no bias in recruitment processes that could affect this study.
Ethics oversight	All subjects provided written informed consent. This study was approved by the Human Genome, Gene Analysis Research Ethics Committee of the University of Tokyo (G0910-(32)) and the Research Ethics Committee of Tokyo Metropolitan Institute of Medical Science (21-10).

Note that full information on the approval of the study protocol must also be provided in the manuscript.
